# Blood Flow Restriction Therapy Stimulates Intercellular Mitochondria Transfer and Improves Muscle Regeneration and Shoulder Function in a Murine Rotator Cuff Injury Model

**DOI:** 10.1177/03635465261424875

**Published:** 2026-03-08

**Authors:** Nesa Milan, Aboubacar Wague, Luke Sang, Alex Youn, Ryan Sadjadi, Yusef Samimi, Cristhian Montenegro, Miguel Lizarraga, Justin Lau, Allan I. Basbaum, Michael R. Davies, Hubert T. Kim, Brian T. Feeley, Jarret A.P. Weinrich, Xuhui Liu

**Affiliations:** * Department of Orthopaedic Surgery, University of California, San Francisco, California, USA; † San Francisco Veterans Affairs Health Care System, San Francisco, California, USA; ‡ University of California, San Francisco, School of Medicine, San Francisco, California, USA; § University of Pittsburgh School of Medicine, Pittsburgh, Pennsylvania, USA; ‖ Department of Anatomy, University of California, San Francisco, California, USA; ¶ Department of Anesthesia and Perioperative Care, University of California, San Francisco, California, USA; Investigation performed at San Francisco Veterans Affairs Health Care System, San Francisco, California, USA

**Keywords:** rotator cuff tears, blood flow restriction, fibro-adipogenic progenitors, mitochondrial transfer

## Abstract

**Background:**

Rotator cuff (RC) tears are among the most common causes of shoulder dysfunction in sports medicine. Muscle atrophy and degeneration are important risk factors for RC tendon retearing and suboptimal recovery of shoulder function after tendon repair. Although blood flow restriction (BFR) can stimulate muscle regeneration after lower extremity trauma and anterior cruciate ligament reconstruction, the mechanisms that underlie BFR remain unknown, and its application to RC tears has not yet been explored.

**Hypothesis:**

The authors hypothesized that BFR induces transfer of mitochondria from intramuscular fibro-adipogenic progenitors (FAPs) to myocytes, enhances muscle regeneration, and improves shoulder function after RC injury.

**Study Design:**

Controlled laboratory study.

**Methods:**

To assess mitochondrial transfer after BFR, the authors used Prrx1-Cre/MitoTag reporter mice, in which FAP mitochondria are labeled. Mice underwent unilateral forelimb BFR, and supraspinatus (SS) muscles were collected at baseline and days 1, 2, 3, 5, and 7 for histology. To model massive RC tears, mice received unilateral SS and infraspinatus tendon transection with denervation (TT+DN) and then were randomized to a BFR (every 3 days) or control group. At 2 or 6 weeks after surgery, SS muscles were analyzed for mitochondrial transfer, fiber size, and fiber-type distribution. Additionally, forelimb gait and weightbearing were captured using the Blackbox system.

**Results:**

BFR was associated with increased FAP-mediated mitochondrial transfer in healthy SS muscle as early as 1 day after BFR treatment and lasted for up to 3 days after BFR. The authors observed an enhanced effect of BFR-induced FAP mitochondrial transfer in SS muscle after RC injury, compared with the control, at both 2 and 6 weeks after TT+DN. BFR-treated mice had significantly reduced muscle atrophy, fatty infiltration, and fibrosis after RC injury. They also observed a significant improvement in forepaw weightbearing ratio and ipsilateral forepaw stride length at 6 weeks after injury in BFR-treated mice compared with controls.

**Conclusion:**

BFR significantly improves muscle quality and shoulder function after RC injury. These effects occur alongside increased mitochondrial transfer from FAPs to myocytes.

**Clinical Relevance:**

Understanding the mechanism of BFR by which BFR enhances muscle regeneration could pave the way for its use as a novel rehabilitation strategy to improve recovery in patients with RC injuries and other muscle-related conditions.

The group of muscles and tendons that comprise the rotator cuff (RC) are critical for the maintenance of shoulder stability and mobility. Composed of 4 main muscles (ie, supraspinatus [SS], infraspinatus, teres minor, and subscapularis), the RC provides both dynamic stabilization of the glenohumeral joint and facilitates a wide range of shoulder movements.^43,59^ Up to 20% of patients >50 years of age have evidence of symptomatic RC tears, with this rate increasing to 49% in patients >70 years.^54^ Individuals with RC tears often experience considerable shoulder pain, which can severely limit their range of motion and the ability to perform basic daily activities, such as reaching, lifting, and personal grooming. Movements that require overhead lifting or external rotation can also exacerbate RC pain, leading to challenges not only in recreational activities but also in occupational tasks.^1,19,42,52^ In the context of an aging patient population in the United States, this condition presents an increasing clinical and economic burden, with an annual medical cost between $1.2 and $1.6 billion (USD).^7,9^ A critical obstacle in the management of RC tears is postinjury muscle degeneration, consisting of muscle atrophy, fatty infiltration (FI), and fibrosis, all of which have been directly linked to higher retear rates and poor outcomes after surgical repair.^12,22,23^ There is currently a lack of effective treatment approaches to enhance muscle quality after RC tears.

Blood flow restriction (BFR) is a therapeutic physical intervention involving the temporary restriction of blood flow to a targeted muscle; this application can induce muscle growth and mitigate muscle atrophy.^10^ Recent studies have also demonstrated that BFR, coupled with low training loads, elicits skeletal muscle hypertrophy and strength gains.^10,41^ This finding has significant implications for rehabilitation practices, particularly for older populations and individuals recovering from musculoskeletal injuries.^10,25^ In orthopaedics, BFR has proven to be beneficial in the rehabilitation of individuals with conditions ranging from fracture healing to tendon and ligament injuries (eg, distal radius fracture, post–anterior cruciate ligament [ACL] reconstruction).^19,26,32,55^ BFR may also benefit postoperative analgesia and pain management.^53^ However, the exact therapeutic mechanisms through which BFR drives functional recovery remain unclear.

Fibro-adipogenic progenitors (FAPs) are a group of heterogeneous, resident skeletal muscle stem cells that have dual and opposing roles in maintaining muscle quality. Depending on the local environment, FAPs can promote both muscle degeneration and regeneration.^1^ In conditions of chronic muscle damage, FAPs differentiate into adipocytes and fibroblasts, contributing to postinjury FI and intramuscular fibrosis, respectively.^14,15,17^ Under pro-regenerative conditions, FAPs can also adopt a beige fat phenotype, a highly metabolic active cell type with high mitochondria content that contributes to muscle regeneration after RC tears.^49,51^ Recent studies have reported that beige FAPs donate mitochondria to recipient myogenic cells, increasing their mitochondrial content and oxidative capacity, thereby supporting muscle regeneration after RC injury.^11^ Preconditioning of muscle with BFR drives FAPs toward beige fat differentiation,^58^ suggesting a potential mechanism by which BFR may facilitate mitochondrial transfer and functional recovery.

Clinically, low-load BFR training has also been shown to improve shoulder internal rotation strength and biceps hypertrophy in patients with RC tendinopathy,^32^ supporting its potential therapeutic role in upper extremity rehabilitation.

Because of the success of BFR in ameliorating molecular and functional deficits in RC tendinopathy and other orthopaedic injury models, in this study, we sought to test the role of BFR in improving RC muscle quality and shoulder function in a murine RC injury model. We tested the hypothesis that after RC injury, BFR would (1) induce mitochondrial transfer from FAPs to myocytes, (2) improve muscle regeneration, and, most importantly, (3) restore shoulder function.

## Methods

### FAP Mitochondria Reporter Mouse Model

All animal procedures were approved by our Institutional Animal Care and Use Committee and performed under institutional review board protocol. Prrx1-Cre/MitoTag FAP-specific mitochondria reporter mice were generated by crossing Prrx1-Cre (Jax No. 005584) and MitoTag (Jax No. 032290) mice. Prrx1 serves as a key mesenchymal transcription factor and a reliable marker for FAPs in muscle tissues.^31,46^ Prrx1-Cre/MitoTag mouse FAPs express a green fluorescent protein (GFP) reporter that localizes to the outer membrane of their mitochondria, thus serving as FAP-derived mitochondria reporter mice.^3^ Mice were studied at the age of 3 months. All mice were maintained on a standard chow diet and kept in an environment with 12-hour light and dark cycles.

### BFR Model

**Figure 1.**
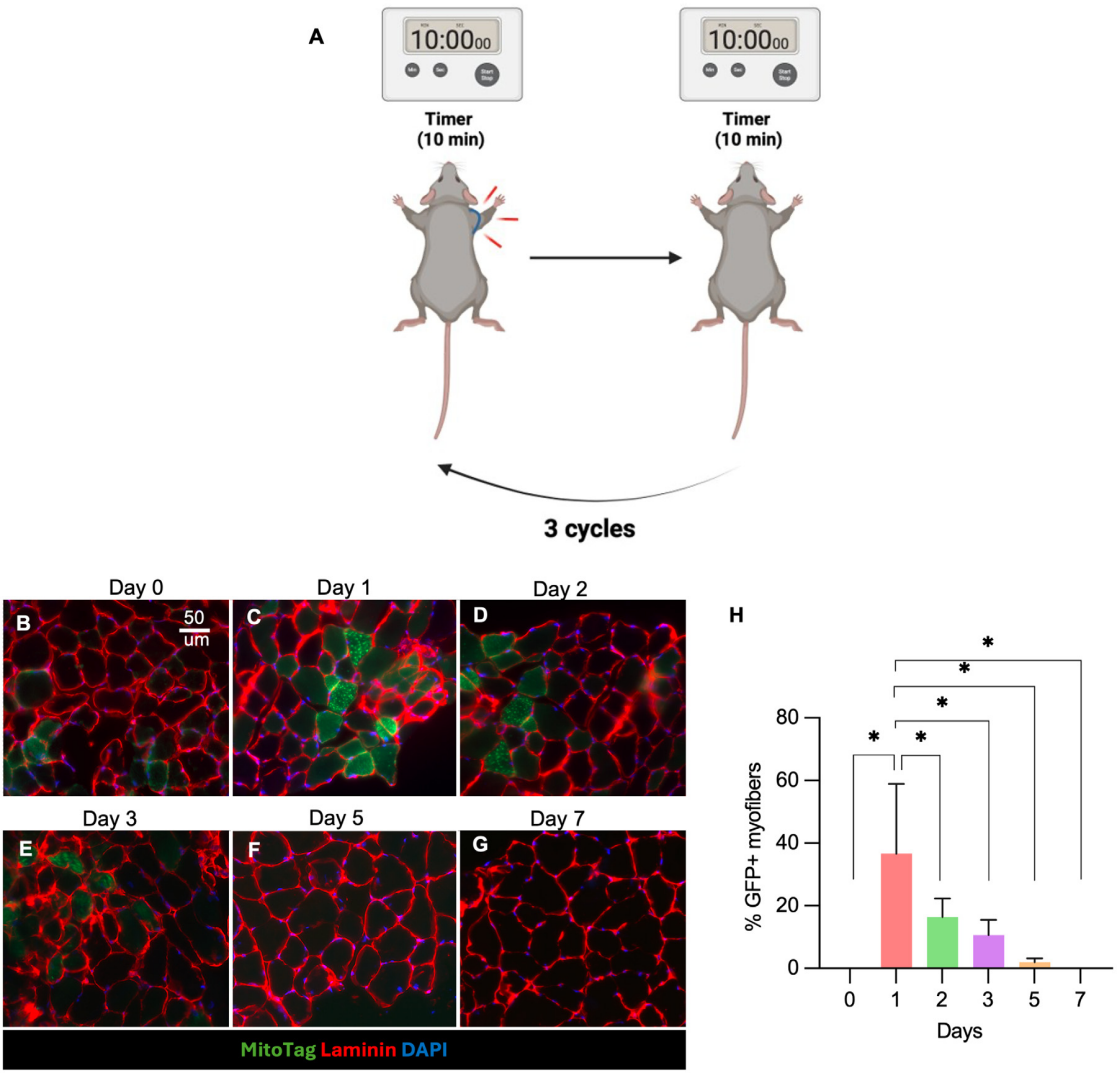
(A) Schematic illustrating the unilateral forelimb blood flow restriction (BFR) protocol. (B-G) Representative histological images of supraspinatus muscle from FAP mitochondria reporter mice at baseline and at various days after ipsilateral arm BFR (scale bar, 50 µm). Mitochondria transferred from FAPs to myofibers are visualized by the MitoTag–green fluorescent protein (GFP) reporter. (H) Quantification of FAP-derived mitochondrial transfer at each time point after BFR, expressed as the percentage of GFP+ mitochondria-containing myofibers out of the total myofibers.

First, mice were anesthetized with 1% to 5% isoflurane in oxygen. Next, unilateral forelimb BFR was performed on 18 Prrx1-Cre/MitoTag mice for histological studies and 12 C57BL/6J mice for functional assessments. To perform BFR, we applied a tourniquet to the ipsilateral forelimb adjacent to the shoulder using a 4.0-ounce orthodontic rubber band for 10 minutes, followed by a 10-minute break for 3 cycles (Figure 1). To evaluate the temporal persistence of BFR-induced effects, mice were euthanized at baseline before BFR and at 1, 2, 3, 5, or 7 days after BFR, and the ipsilateral SS muscles were harvested (n = 18).

To simulate a massive RC tear, we made a unilateral transection of the SS and infraspinatus tendons combined with suprascapular nerve denervation (TT+DN) as previously described.^40^ Briefly, under isoflurane anesthesia, a longitudinal skin incision was made over the shoulder, and the deltoid was bluntly retracted to expose the RC tendons. The SS and infraspinatus tendons were transected at their insertion on the humerus. To prevent reinnervation, we transected a 2- to 3-mm segment of the suprascapular nerve proximal to the suprascapular notch. Mice were then randomized into the BFR or control group (6 per group). Mice in the BFR group received ipsilateral forelimb BFR once every 3 days; mice in the control group underwent the same anesthesia procedure without BFR treatment. Mice were euthanized at either 2 or 6 weeks after surgery, and bilateral SS muscles were harvested for histological analysis.

### Muscle Histology Quantification and Image Capture

The SS muscles were harvested from the Prrx1-Cre/MitoTag mice and mounted on cork disks with 10% tragacanth gum in water and flash-frozen in liquid nitrogen–cooled isopentane. Frozen SS muscle was sectioned at a thickness of 10 μm using a cryostat. All muscles were sectioned at the midbelly region of the SS muscle, and 3 representative sections per muscle were analyzed to ensure consistency across samples for histological and quantitative analyses. Muscle sections were fixed with 4% paraformaldehyde for 10 minutes and washed 3 times for 5 minutes with phosphate-buffered saline (PBS). Then, sections were blocked with 5% bovine serum albumin in PBS (Thomas Scientific) for 1 hour at room temperature and incubated with an anti-laminin primary antibody (Abcam; ab11576, 1:200) overnight at 4°C. The next day, sections were washed again with PBS, 3 times for 5 minutes, and incubated with a secondary antibody, donkey anti-rat Alexa Fluor 647 (Abcam; ab150155, 1:200). Lastly, the slides were washed 3 more times with PBS for 5 minutes and then mounted with coverslips using Fluoromount-G with DAPI (Invitrogen). Mitochondrial transfer was quantified as the number of GFP^+^ fibers in muscle sections from Prrx1-Cre/MitoTag mice. To assess the degree of FI and fibrosis, sections were stained with Oil Red O (Sigma Aldrich) and Masson Trichrome (American Mastertech), respectively, per the manufacturers’ instructions, as previously described.^33^ For quantification, the percentage of FI and fibrotic area was calculated as the ratio of positively stained area, defined using consistent color thresholding of red Oil Red O signal (fat) or blue Trichrome collagen (fibrosis), to total tissue area in each section using FIJI (ImageJ; National Institutes of Health). Three representative fields per section were analyzed per sample.

For muscle fiber type analysis, we immunostained for myosin heavy chain (MyHC) isoforms on frozen sections. After blocking with 5% bovine serum albumin in PBS (Thomas Scientific), sections were incubated overnight at 4°C with primary antibodies for MyHC isoforms: type I (BA-D5), type IIA (SC-71), and type IIB (BF-F3), all from the Developmental Studies Hybridoma Bank. The following day, sections were washed with PBS and incubated with Alexa Fluor–conjugated secondary antibodies: Alexa Fluor 350 anti-mouse IgG2b (type I), Alexa Fluor 594 anti-mouse IgG1 (type IIA), and Alexa Fluor 647 anti-mouse IgM (type IIB) (Invitrogen; 1:200 dilution) for 1 hour at room temperature in the dark. Type IIX fibers were not directly stained but were identified by exclusion, defined as fibers lacking immunoreactivity for type I, IIA, and IIB, as previously described.^4^ Finally, sections were washed, mounted with Fluoromount-G containing DAPI (Invitrogen), and coverslipped for imaging. All images were captured with a Keyence BZ-X fluorescence microscope and analyzed using FIJI. Myofiber types were identified by immunolabeling and quantified as a percentage of total fibers across multiple sections per sample. All quantifications were performed by researchers (A.Y., R.S., Y.S., M.L.) blind to the treatment group.

### Longitudinal Assessment of Murine Shoulder Function

**Figure 2.**
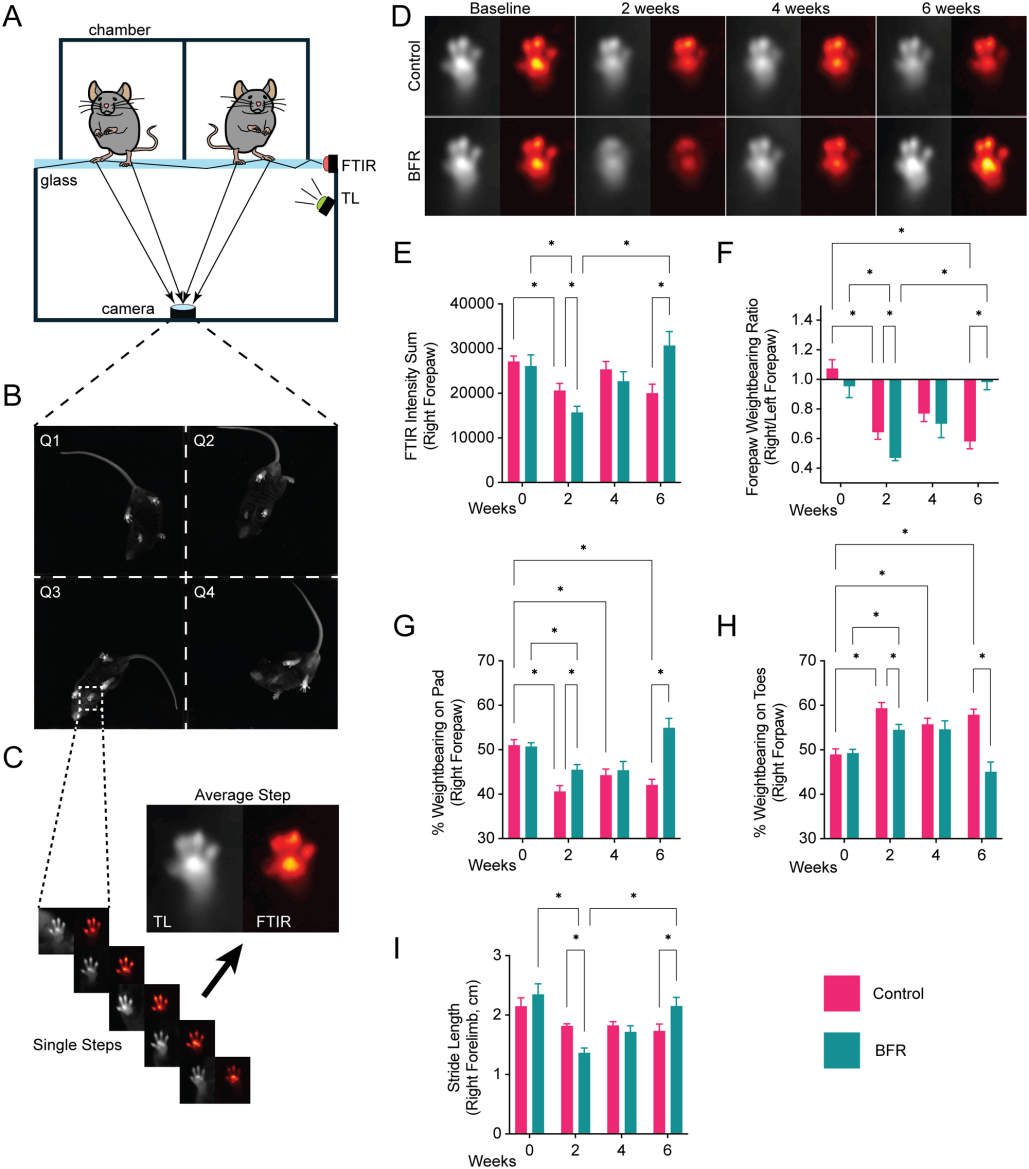
(A-C) Blood flow restriction (BFR) improves forelimb function after rotator cuff (RC) injury. Blackbox setup schematic: 4-chamber arena, near-infrared camera, transillumination (TL), and frustrated total internal reflectance (FTIR) LED strips. (D) Representative plantar pressure heatmap of the ipsilateral (right) mouse forepaw, with red/orange colors denoting higher applied pressure, demonstrating decreased weightbearing after RC injury and improved weightbearing after BFR treatment. (E) Sum of the FTIR intensity in the right forepaw. (F) Quantification of forepaw weightbearing as a right-to-left ratio, where 1.0 indicates equal weight distribution between limbs. Percentage of total weightbearing borne by the (G) pad and (H) toes of the right forepaw. (I) Quantification of right forepaw stride length. *P < .05.

We used the Blackbox R4 device (Blackbox Bio) to record changes in limb weightbearing and kinematics, as previously described^33^ (Figure 2A). Behavior was captured at baseline and 2, 4, and 6 weeks postoperatively for BFR and control mice. Briefly, each mouse was placed into 1 of 4 dark acrylic chambers, where they were allowed to freely roam. The acrylic chamber sits atop borosilicate glass, under which is a high temporal and spatial resolution near-infrared (NIR) camera that records animal behavior. Mice were illuminated by 2 sets of NIR LED lights: (1) transillumination (TL) light to visualize overall mouse body pose and (2) frustrated total internal reflectance (FTIR) light to visualize paw contact pressure with the glass floor. To allow for visualization of paw contact alone, TL LEDs were turned on and off in an alternating fashion, with every other frame captured by the camera. FTIR LEDs remained on throughout the entire recording.

The output of a single recording session was 2 videos, one of which was a combination of TL and FTIR signal, and the other the FTIR signal alone. Four-chamber videos were then split using FFmpeg so that each split video consisted of only 1 chamber (eg, mouse). Next, we used machine learning–based video object tracking (DeepLabCut Version 1.5.7) to label mouse landmarks (eg, right forepaw). To measure forepaw weightbearing and kinematics, these labeled landmarks were analyzed using custom-written MATLAB code (MathWorks), as previously described.^34^ Forepaw FTIR intensity was measured as the summed intensity of FTIR signal across the entire forepaw, averaged across all steps identified within a recording (Figure 2C). The forepaw weightbearing ratio is the ratio of injured (right) to uninjured (left) mean forepaw FTIR intensity. The percentage of weightbearing on toes or pad was measured as the percentage of FTIR signal corresponding to the toes and pad within the image over the total FTIR signal for the entire forepaw, multiplied by 100%, which was then averaged across all steps identified within a recording. Lastly, stride length was measured as the distance the forepaw travels during stepping during walking bouts.

### Statistical Analysis

Unpaired *t* tests were used for statistical analysis between BFR and control groups for mice with RC injury. One-way analysis of variance (ANOVA) with the Tukey honestly significant difference post hoc test was used when >2 groups were compared. For behavioral data analyzed using Blackbox, either 2-way ANOVA or linear mixed-effects models were applied, depending on the experimental design. Significance was considered at a *P* value <.05. All data are presented in the form of mean ± standard error. All statistical analyses were computed in GraphPad Prism (Version 10.4).

## Results

### BFR Improves Shoulder Function After RC Injury

We assessed the efficacy of BFR in improving forelimb function in RC-injured mice. To simulate a massive RC tear, wild-type C57BL/6J mice underwent a combined tendon transection and nerve cut (TT+DN), as previously described,^40^ and were then randomized to BFR or control groups. To monitor changes in forelimb function, we used a recently developed behavioral monitoring technology, the Blackbox system,^3^ to capture the naturalistic behaviors of freely moving mice (Figure 2, A and B). To quantify changes in forelimb function, we developed a machine learning–enabled data processing pipeline that extracted forelimb weightbearing and kinematic parameters from behavioral recordings^34^ (see Methods) (Figure 2C).

Our analysis clearly demonstrates that the RC tear model produces quantifiable deficits in forelimb function and that BFR can significantly improve forelimb function (Figure 2, D-I). In control mice without BFR, at 2 weeks after RC injury, we recorded decreased weightbearing on the injured forelimb (Figure 2, D-F), with concomitant changes in the distribution across different parts of the forepaw (ie, toes vs pad) (Figure 2, G and H). These findings include ipsilateral forepaw FTIR intensity (a proxy for contact pressure; 20,631.02 ± 4549.79 vs 15,687.61 ± 3935.28; 6 per group; *P* = .04, 2-way ANOVA), ipsilateral/contralateral weightbearing ratio (1.0 ± 0.1 vs 0.6 ± 0.1; 6 per group; *P* = .005, 2-way ANOVA), and percent weightbearing on the pad (40.63 ± 3.66 vs 45.48 ± 3.40; 6 per group; *P* = .02, 2-way ANOVA) and toes (59.37 ± 3.66 vs 54.52 ± 3.40; 6 per group; *P* = .02; 2-way ANOVA). However, in mice treated with BFR, at 6 weeks after RC injury, we recorded significant functional improvement of weightbearing on the injured forelimb (Figure 2, D-H), including ipsilateral forepaw FTIR intensity (20,005 ± 5762.48 vs 30,709.58 ± 8800.86; 6 per group; *P* = .01, 2-way ANOVA), ipsilateral/contralateral weightbearing ratio (0.58 ± 0.14 vs 0.98 ± 0.14; 6 per group; *P* < .0001, 2-way ANOVA), and percent weightbearing on the pad (42.09 ± 3.56 vs 54.92 ± 6.16; 6 per group; *P* = .0002, 2-way ANOVA) and toes (57.91 ± 3.56 vs 45.08 ± 6.16; 6 per group; *P* = .0002; 2-way ANOVA). When assessing kinematic parameters of the injured forelimb, we detected a transient deficit at 2 weeks in BFR-treated mice compared with controls; however, this effect dissipated by 6 weeks after RC injury in the BFR-treated group (2.2 ± 0.1 cm at 6 weeks vs 1.3 ± 0.1 cm at 2 weeks; 6 per group; *P* < .05, linear mixed-effects model) (Figure 2I).

### BFR Induces Mitochondrial Transfer From FAPs to Myofibers in Healthy SS Muscle

To understand the underlying effect of BFR on FAP-mediated mitochondrial transfer and the duration of its effects, SS muscles from Prrx1-Cre/MitoTag mice were harvested for histology at various time points after BFR. At baseline (day 0), GFP^+^ mitochondria were confined to interstitial FAPs and located outside laminin-bounded myofibers. BFR significantly induced mitochondrial transfer from FAPs to myocytes (Figure 1, A-H). This effect peaked at 1 day after BFR, where a mean of 36.6% ± 11.4% (3 per group; *P* < .05) of myofibers contain GFP^+^ mitochondria transferred from FAPs. This effect lasted up to 3 days after BFR (10.4% ± 3.4%; 3 per group; *P* < .05, ordinary 1-way ANOVA compared with baseline) (Figure 1H).

### BFR Increases FAP-Mediated Mitochondrial Transfer and Reduces Myofiber Atrophy After RC Injury

**Figure 3.**
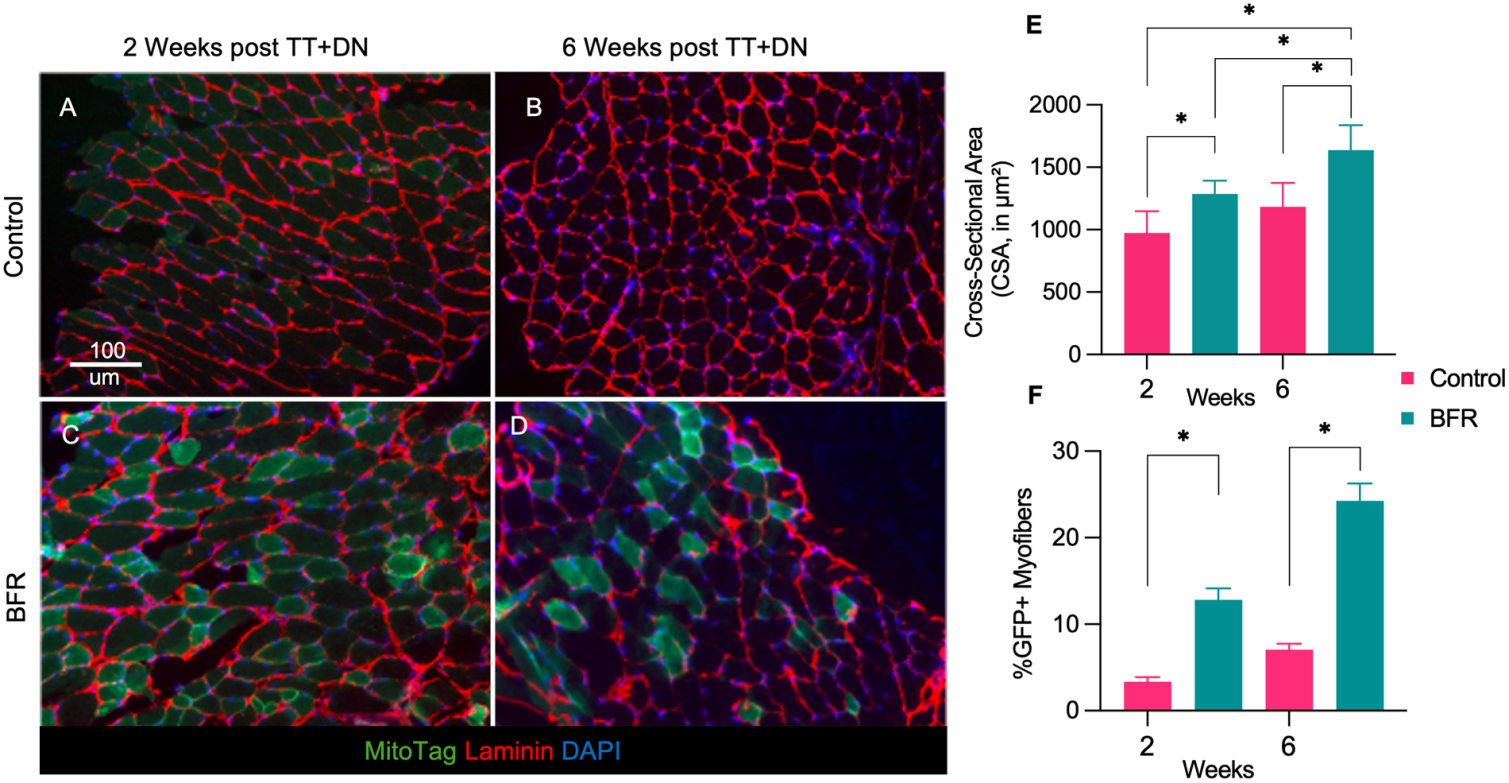
Fibro-adipogenic progenitor (FAP)–mediated mitochondrial transfer and myofiber size in supraspinatus (SS) muscle after blood flow restriction (BFR) and rotator cuff injury. (A-D) Representative histological images of SS muscle from FAP mitochondria reporter mice at baseline and at various days after ipsilateral arm BFR in the Prrx1-Cre/MitoTag reporter mice at 2 and 6 weeks after unilateral SS and infraspinatus tendon transection and denervation (TT+DN), with or without ipsilateral arm BFR (scale bar, 100 µm). (E) Quantification of myofiber cross-sectional area (CSA) in each group. (F) Quantification of mitochondrial transfer postinjury, defined as the percentage of green fluorescent protein–positive (GFP+) myofibers per total myofibers. *P < .05.

Given that BFR improved forelimb function after TT+DN injury, we next examined whether BFR also enhances intercellular mitochondrial transfer and muscle regeneration after RC injury. As we found that the effect of a single episode of BFR lasts up to 3 days after the intervention, mice in the treatment group received BFR every 3 days until they were euthanized at either 2 or 6 weeks after TT+DN. Compared with the mice in the untreated control group, mice that received BFR for 2 weeks after TT+DN had significantly increased mitochondrial transfer from FAPs (12.8% ± 1.3% vs 3.3% ± 0.6%; 6 per group; *P* < .05, ordinary 1-way ANOVA) (Figure 3). We recorded a comparable increase in mitochondrial transfer in the SS muscle of BFR-treated mice compared with the control mice at 6 weeks after TT+DN (25.3% ± 2.0% vs 7.1% ± 0.7%; 6 per group; *P* < .05, ordinary 1-way ANOVA) (Figure 3).

Next, to characterize the effects of BFR on muscle adaptation, we assessed the distribution of myofiber sizes and found that BFR-treated mice had a significantly larger mean cross-sectional area than the control mice (Figure 3E). This effect was present in the SS muscle both 2 weeks (1287.0 ± 42.7 µm^2^ vs 972.4 ± 71.1 µm^2^; 6 per group; *P* < .05, ordinary 1-way ANOVA) and 6 weeks (1637.0 ± 89.3 µm^2^ vs 1183.0 ± 78.0 µm^2^; 6 per group; *P* < .05, ordinary 1-way ANOVA) after BFR.

### Fiber Type Distribution and MitoTag+ Fibers

MHC-IIX and MHC-IIB fibers constitute the majority of the overall myofiber population across all groups and time points, with MHC-IIA fibers present at lower frequencies and MHC-I fibers remaining sparse. This distribution remained consistent across both control and BFR-treated muscles over time.

**Figure 4.**
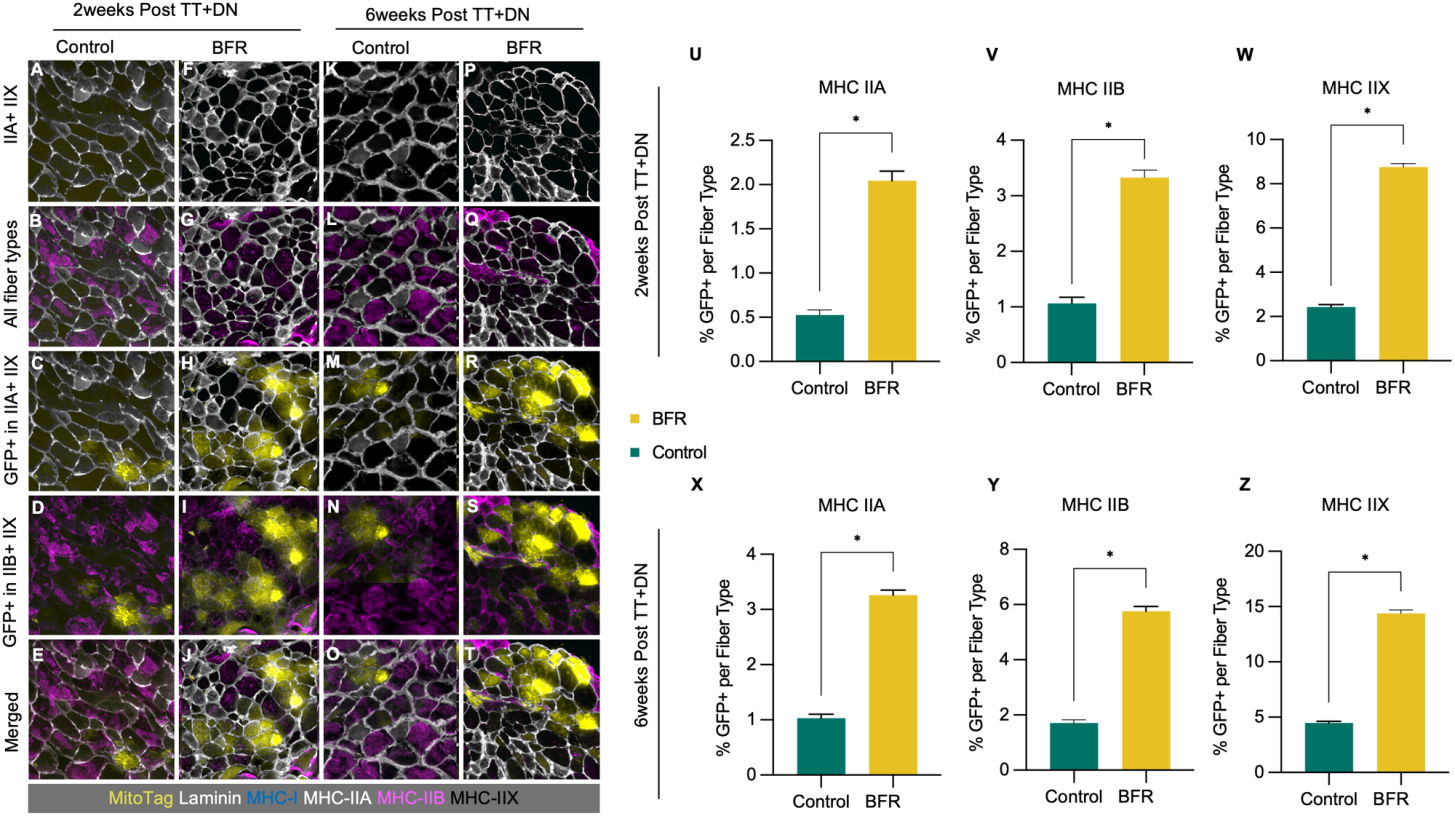
Comparison of muscle fiber type and MitoTag fiber distribution after rotator cuff injury between blood flow restriction (BFR) and control groups. (A-T) Representative muscle fiber histological images of supraspinatus (SS) muscle after unilateral SS and infraspinatus tendon transection and denervation (TT+DN) in Prrx1-Cre/MitoTag mice receiving BFR and control. Muscle fiber types were determined via major histocompatibility complex (MHC) expression staining. (U-Z) Quantification of green fluorescent protein–positive (GFP^+^) fibers per fiber type, calculated as follows: % GFP^+^ per fiber type = (number of GFP^+^ fibers of a given fiber type)/(total fibers of that fiber type) × 100. Graphs show significantly increased mitochondrial transfer in BFR groups across MHC-IIA, IIB, and IIX fibers at both (U-W) 2 weeks and (X-Z) 6 weeks after injury. Data are shown as mean ± SEM. *P < .05 by unpaired Student *t* test.

To assess whether BFR promotes mitochondrial transfer in a fiber type–specific manner after RC injury, we quantified GFP^+^ myofibers in Prrx1-Cre/MitoTag mice at 2 and 6 weeks after TT+DN (Figure 4). At both 2 and 6 weeks, respectively, the percentage of GFP^+^ fibers was significantly higher in the BFR group across MHC-IIA (control: 0.53% ± 0.09%, BFR: 2.04% ± 0.14%, 6 per group, *P* < .01, Student *t* test at 2 weeks; control: 1.03% ± 0.06%, BFR: 3.26% ± 0.12%, 6 per group, *P* < .01, Student *t* test at 6 weeks), MHC-IIB (control: 1.06% ± 0.14%, BFR: 3.33% ± 0.18%, 6 per group, *P* < .01, Student *t* test at 2 weeks; control: 1.71% ± 0.12%, BFR: 5.75% ± 0.22%, 6 per group, *P* < .01, Student *t* test at 6 weeks), and MHC-IIX (control: 2.43% ± 0.10%, BFR: 8.76% ± 0.12%, 6 per group, *P* < .01, Student *t* test at 2 weeks; control: 4.47% ± 0.21%, BFR: 14.38% ± 0.23%, 6 per group, *P* < .01; Student *t* test at 6 weeks) fibers. No GFP+ labeling was observed in MHC-I fibers across any group or time point.

### BFR Reduces FI and Fibrosis in the SS Muscle After RC Injury

**Figure 5.**
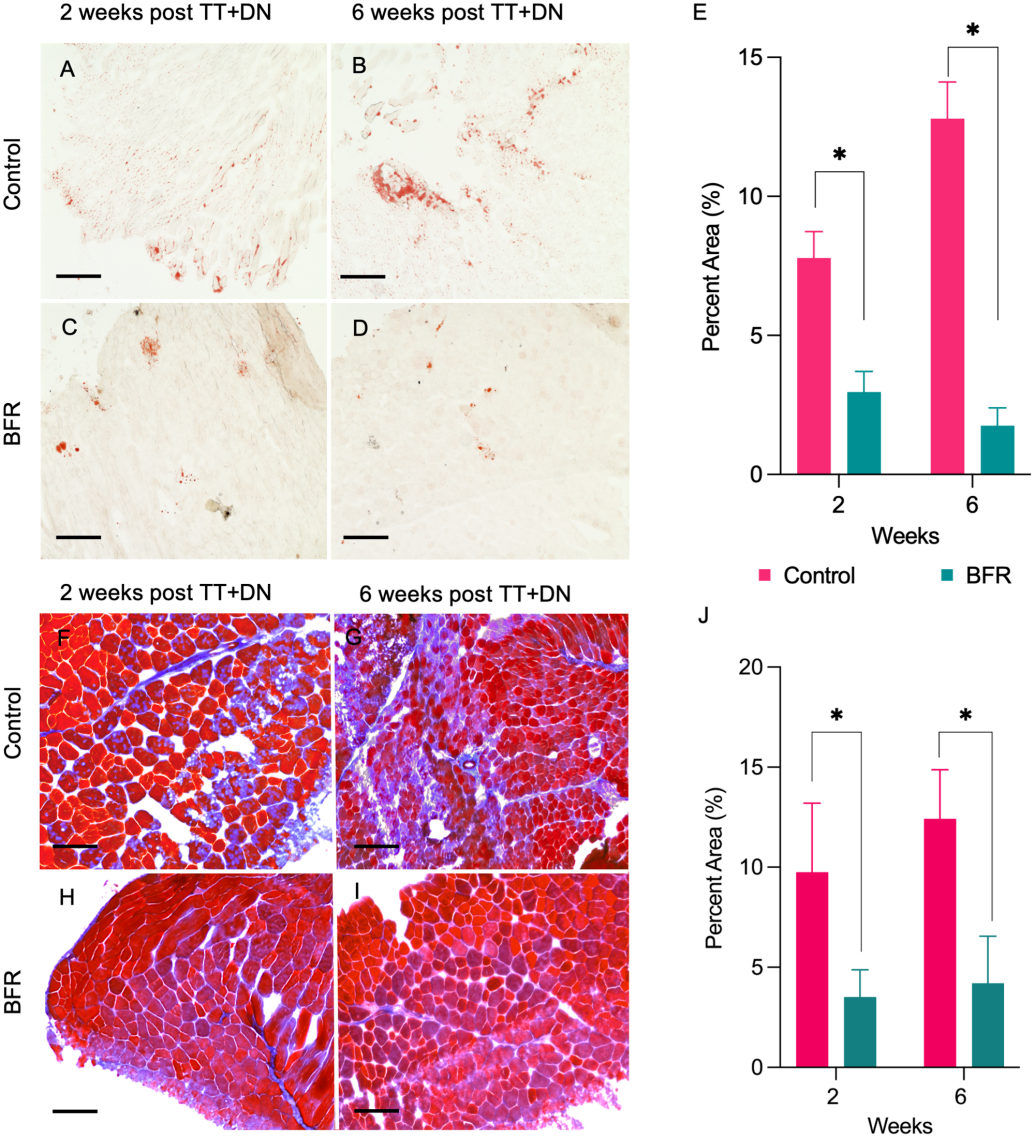
Effects of blood flow restriction (BFR) on fatty infiltration (FI) and fibrosis after rotator cuff injury. (A-D) Representative histological images of supraspinatus (SS) muscle Oil Red O staining to determine the amount of degenerative FI (scale bars, 100 μm) in control and BFR-treated mice at 2 and 6 weeks after unilateral SS and infraspinatus tendon transection and denervation (TT+DN). (E) Quantification of the percent area of FI (positive stain signal per total section area). (F-I) Representative histological images of SS muscle Masson Trichrome staining to determine the amount of fibrosis development (scale bars, 100 μm) in control and ipsilateral arm BFR-treated mice at 2 and 6 weeks after TT+DN. (J) Quantification of percent area of fibrosis (positive stain signal per total section area) *P < .05.

After determining that BFR increases FAP mitochondrial transfer and reduces muscle atrophy after RC injury, we investigated its ability to mitigate other adverse postinjury outcomes, namely, muscle FI (Figure 5) and fibrosis. Muscle remodeling was assessed using Oil Red O staining to visualize intramuscular adipose infiltration and Masson Trichrome staining to detect endomysial collagen deposition. Low-magnification images of these stains for all groups are provided in Appendix Figure A1 (available in the online version of this article) to illustrate overall tissue architecture. Oil Red O staining confirmed that BFR significantly reduces FI at both 2 weeks (3.0% ± 0.7% vs 7.8% ± 1.0%; 6 per group; *P* < .05, 1-way ANOVA) and 6 weeks (1.8% ± 0.6% vs 12.8% ± 1.3%; 6 per group; *P* < .05, 1-way ANOVA) after RC injury. Additionally, in BFR-treated mice, we recorded a significant decrease in fibrotic area in SS muscles compared with control mice, both at 2 weeks (3.5% ± 0.8% vs 9.7% ± 1.7%; 6 per group; *P* < .05, 1-way ANOVA) and 6 weeks (4.2% ± 1.4% vs 12.4% ± 1.4%; 6 per group; *P* < .05, 1-way ANOVA) after RC injury.

## Discussion

In this study, we demonstrated the effect of BFR on reducing RC muscle atrophy, fibrosis/FI, and improving shoulder function after RC tear. These improvements are concomitant with increased promyogenic mitochondrial transfer in a murine model of tendon TT+DN. Importantly, these histological improvements were accompanied by improved forelimb mobility, as evidenced by increased forepaw weightbearing and stride length. These findings suggest that BFR can significantly enhance muscle quality and improve shoulder function after RC injury and are consistent with an association between BFR treatment and increased GFP^+^ signal.

FAPs are central mediators in the degenerative processes after RC injuries. After tendon injury, FAPs become activated, and in the presence of pro-fibrotic and pro-adipogenic signals, such as transforming growth factor-β, FAPs differentiate into fibroblasts and adipocytes. This differentiation leads to pronounced fibrosis and FI, further degrading muscle quality and impairing repair outcomes.^16,30,35,47^ The persistence of these cell phenotypes is detrimental; in animal models, prolonged FAP survival and accumulation are associated with muscle degeneration.^35^ On the other hand, FAPs also possess latent regenerative potential. Under specific microenvironmental conditions, such as exposure to IL-33 or β-adrenergic stimulation, FAPs can differentiate into a beige adipocyte phenotype, characterized by increased mitochondrial content and enhanced secretion of promyogenic growth factors.^50^ This process, known as FAP browning, has been shown to reduce fibrosis and FI and to enhance regenerative potential, as demonstrated in ischemia-reperfusion and RC injury models.^58^ This beige FAP phenotype has been shown, in preclinical studies, to create a pro-regenerative niche that supports skeletal muscle repair and promotes myogenesis through the activation and proliferation of satellite cells.^50,57^ Furthermore, therapeutic strategies that target FAP plasticity, aiming either to inhibit their differentiation into adipocytes and fibrotic cells or to promote their conversion into beige promyogenic cells, are being developed as potential therapeutics to improve muscle quality after RC tear.^57^

Horizontal mitochondrial transfer has been described in a variety of settings between mesenchymal stem cells (MSCs) and oxidatively damaged recipient cells^6,29,36,48^ and is thought to be a mechanism by which MSCs help restore tissue respiration and promote regeneration.^24^ We recently found that FAPs donate mitochondria to myogenic recipient cells within the injured RC and that this process is amplified with beige FAP differentiation, promoting enhanced muscle regeneration.^11^ Here, we found that BFR is associated with increased FAP-mediated mitochondrial transfer both in healthy muscle and after RC injury. Taken together, these findings are consistent with mitochondrial transfer as a potential contributing process in the BFR-associated regenerative response.

These findings likely represent interconnected FAP-mediated processes. Previous work from our group demonstrated that preconditioning activates β3-adrenergic signaling to induce FAP browning, which promotes muscle regeneration and reduces fibrosis after ischemia-reperfusion injury.^58^ Additionally, Chi et al^11^ showed that FAPs directly transfer mitochondria to regenerating myofibers, enhancing oxidative metabolism and muscle repair after RC injury. In our study, we examine this process and show that BFR further augments this endogenous mitochondrial transfer.

Together, these studies suggest that BFR may integrate both pathways, FAP browning, which limits deleterious differentiation into fibroblasts and adipocytes, and FAP-derived mitochondrial transfer, which may enhance cellular metabolism and contribute to increases in myofiber cross-sectional area.

Beyond these observations, accumulating evidence highlights the importance of mitochondrial quality and bioenergetic homeostasis in muscle regeneration. Mitochondrial dysfunction, impaired oxidative capacity, and disrupted network dynamics are hallmarks of injured skeletal muscle, and restoration of mitochondrial function has been shown to be essential for effective repair.^2,18^ Recent high-profile studies have demonstrated that mitochondrial transfer from MSCs can mediate tissue repair across diverse organ systems, acting not only as a metabolic supplement but also as a stimulus for cell signaling, mitophagy, and adaptive remodeling.^5,39^ These findings support the plausibility that FAP-derived mitochondria may influence regenerating myofibers through metabolic and signaling pathways relevant to recovery after RC injury. The reductions in FI and fibrosis after BFR align more closely with the known effects of FAP browning, which shifts FAP fate away from fibro-adipogenic differentiation and toward a pro-regenerative state.

Although we observe increased mitochondrial transfer from FAPs to myofibers in BFR-treated mice, how recipient muscle cells use the transferred mitochondria remains unknown. Previous studies have reported that transferred mitochondria can enhance oxidative metabolism, ATP production, and overall bioenergetic capacity in damaged cells.^8,45^ Mitochondrial transfer may also activate intracellular signaling pathways. Recent work in endothelial cells suggests that transferred mitochondria can serve as signaling pathways that initiate mitophagy in recipient cells, thereby promoting cellular adaptation and tissue repair, even when the mitochondria are not functionally integrated.^39^ These findings suggest that mitochondrial uptake serves as both a source of metabolic support and a stimulus for adaptive cellular remodeling. In skeletal muscle, similar mechanisms may initiate the regenerative effects of BFR-induced mitochondrial transfer, potentially through mitophagy, mitochondrial biogenesis, or metabolic reprogramming that facilitates muscle repair.

The observed reduction in myofiber atrophy after BFR in the SS muscle after RC injury aligns with existing literature demonstrating BFR’s efficacy in promoting muscle hypertrophy. A previous study showed that BFR applied during low-load resistance training induces similar increases in muscle fiber cross-sectional area compared with high-load resistance training, suggesting that BFR can effectively stimulate muscle growth with reduced mechanical load.^38^ Additionally, a systematic review indicated that low-load BFR training can be an effective method to elicit increases in muscle size, with most research showing similar whole-muscle development of the extremities compared with high-load training.^44^ The underlying mechanisms by which BFR promotes hypertrophy are thought to include increased metabolic stress, accumulation of metabolites, and enhanced muscle fiber recruitment, all of which contribute to muscle growth.^27^ In the context of RC injury, where muscle atrophy and degeneration are prevalent, the application of BFR may serve as a valuable therapeutic strategy to counteract these deleterious effects and facilitate muscle regeneration.

Our behavioral studies showed significant improvements in both forepaw weightbearing ratio and ipsilateral stride length at 6 weeks in the BFR-treated group. These functional enhancements align with our histological findings, which showed increased myofiber cross-sectional area, elevated mitochondrial transfer from FAPs to myofibers, and significant reductions in both FI and fibrosis within the injured SS muscle. Notably, BFR-treated mice demonstrated progressive and significant improvements in forelimb function over time, indicating that BFR not only promotes structural preservation but also translates into measurable functional improvements in naturalistic behavior and motor performance.

These findings are consistent with and extend previous preclinical work in a bilateral ACL reconstruction rat model, where BFR preserved muscle fiber size and reduced postsurgical atrophy.^20^ Additionally, in clinical studies involving patients undergoing rehabilitation after ACL reconstruction, low-load resistance training combined with BFR significantly improved muscle strength, reduced disuse atrophy, and enhanced return to function.^21,28,56^

Multiple randomized controlled clinical trials and meta-analyses also found that BFR applied to the proximal thigh during rehabilitation after ACL reconstruction can improve muscle strength, reduce atrophy, and enhance functional recovery in the early postoperative period.^13,28,37^ Together, these results underscore the functional and translational relevance of BFR across species and injury models. Our study further validates these findings by integrating advanced behavioral analysis with cellular and tissue-level outcomes, establishing a comprehensive framework to evaluate BFR as a regenerative strategy for musculoskeletal injuries.

### Limitations

This study does have some limitations. One remaining question is whether BFR exerts its regenerative effects in the shoulder through systemic mechanisms, such as modifying circulating cytokines or vascular-derived signals. Although we did not directly assess systemic factors, the observed increase in mitochondrial transfer and promyogenic features, such as larger myofiber cross-sectional area and reduced FI and fibrosis, suggests the involvement of paracrine or systemic signaling. To better define the mechanisms involved, our future studies will profile serum cytokines and vascular signaling after BFR. Also, only a single BFR application protocol was evaluated, based on preliminary data. Exploring different frequencies and durations of BFR treatment may help optimize its effects on muscle quality and shoulder function. Additionally, we conducted the study in relatively young animals. We did not perform a comprehensive pain-related behavioral assessment, and the effects of BFR were not examined in the context of RC repair. To assess the potential of BFR to enhance recovery after surgical intervention, our future studies will address this limitation by applying BFR in a postrepair model.^17^

## Conclusion

BFR is associated with increased FAP-mediated mitochondrial transfer to myocytes in healthy SS muscle. After RC injury, BFR reduces muscle atrophy and degeneration and improves shoulder function. These findings were concomitant with enhanced mitochondria transfer from FAPs to myocytes in the RC muscle. Taken together, this preclinical study suggests that upper limb BFR is a promising rehabilitation therapy to improve shoulder function in patients with RC tears.

## Supplemental Material

sj-pdf-1-ajs-10.1177_03635465261424875 – Supplemental material for Blood Flow Restriction Therapy Stimulates Intercellular Mitochondria Transfer and Improves Muscle Regeneration and Shoulder Function in a Murine Rotator Cuff Injury Model Supplemental material, sj-pdf-1-ajs-10.1177_03635465261424875 for Blood Flow Restriction Therapy Stimulates Intercellular Mitochondria Transfer and Improves Muscle Regeneration and Shoulder Function in a Murine Rotator Cuff Injury Model by Nesa Milan, Aboubacar Wague, Luke Sang, Alex Youn, Ryan Sadjadi, Yusef Samimi, Cristhian Montenegro, Miguel Lizarraga, Justin Lau, Allan I. Basbaum, Michael R. Davies, Hubert T. Kim, Brian T. Feeley, Jarret A.P. Weinrich and Xuhui Liu in The American Journal of Sports Medicine
