# Supplementary material for: Blood Flow Restriction Therapy Stimulates Intercellular Mitochondria Transfer and Improves Muscle Regeneration and Shoulder Function in a Murine Rotator Cuff Injury Model
Source: Am J Sports Med. 2026 Mar 8;54(5):1114–25. doi: 10.1177/03635465261424875 (PMC13031367; doi:10.1177/03635465261424875)
Supplement: sj-pdf-1-ajs-10.1177_03635465261424875 – Supplemental material for Blood Flow Restriction Therapy Stimulates Intercellular Mitochondria Transfer and Improves Muscle Regeneration and Shoulder Function in a Murine Rotator Cuff Injury Model [file sj-pdf-1-ajs-10.1177_03635465261424875.pdf]

Blood Flow Restriction Therapy Stimulates Intercellular Mitochondria Transfer and Improves Muscle Regeneration and Shoulder Function in a Murine Rotator Cuff Injury Model

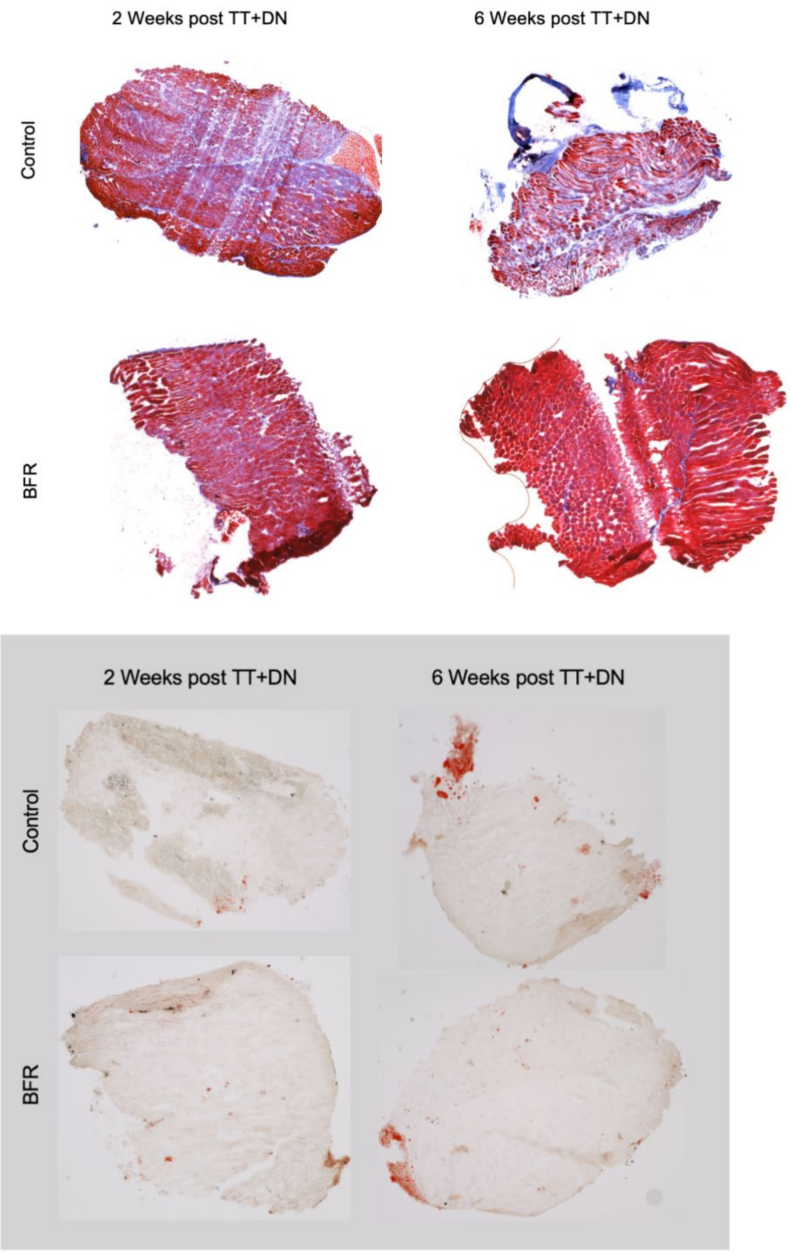

Figure A1.

Low-magnification Masson Trichrome and Oil Red O images of supraspinatus muscles from control and BFR-treated mice at 2 and 6 weeks post-TT+DN. Trichrome highlights collagen deposition (blue), and Oil Red O marks lipid infiltration (red), providing an overview of fibrosis and fatty infiltration across groups.

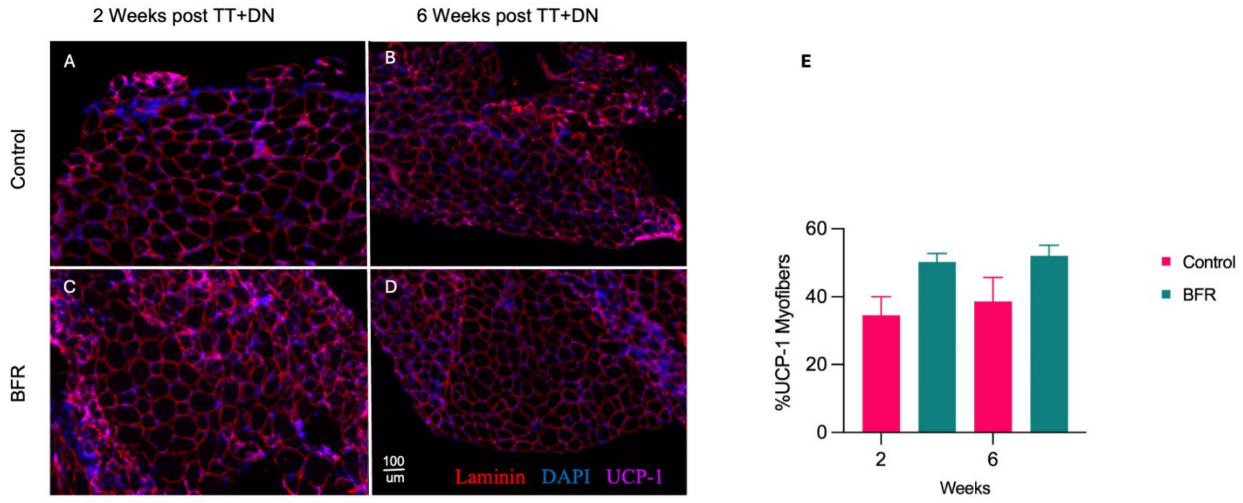

Figure A2. UCP-1 expression after RC injury with or without BFR.

Representative supraspinatus sections at 2- and 6-weeks post-TT+DN stained for laminin (red), DAPI (blue), and UCP-1 (magenta). UCP-1$^+$ cells were present in all groups, with qualitatively stronger staining in BFR-treated muscles. Quantification of UCP-1$^+$ myofibers (E) showed higher mean values in BFR mice, although differences were not statistically significant. Scale bar = 100 μm.

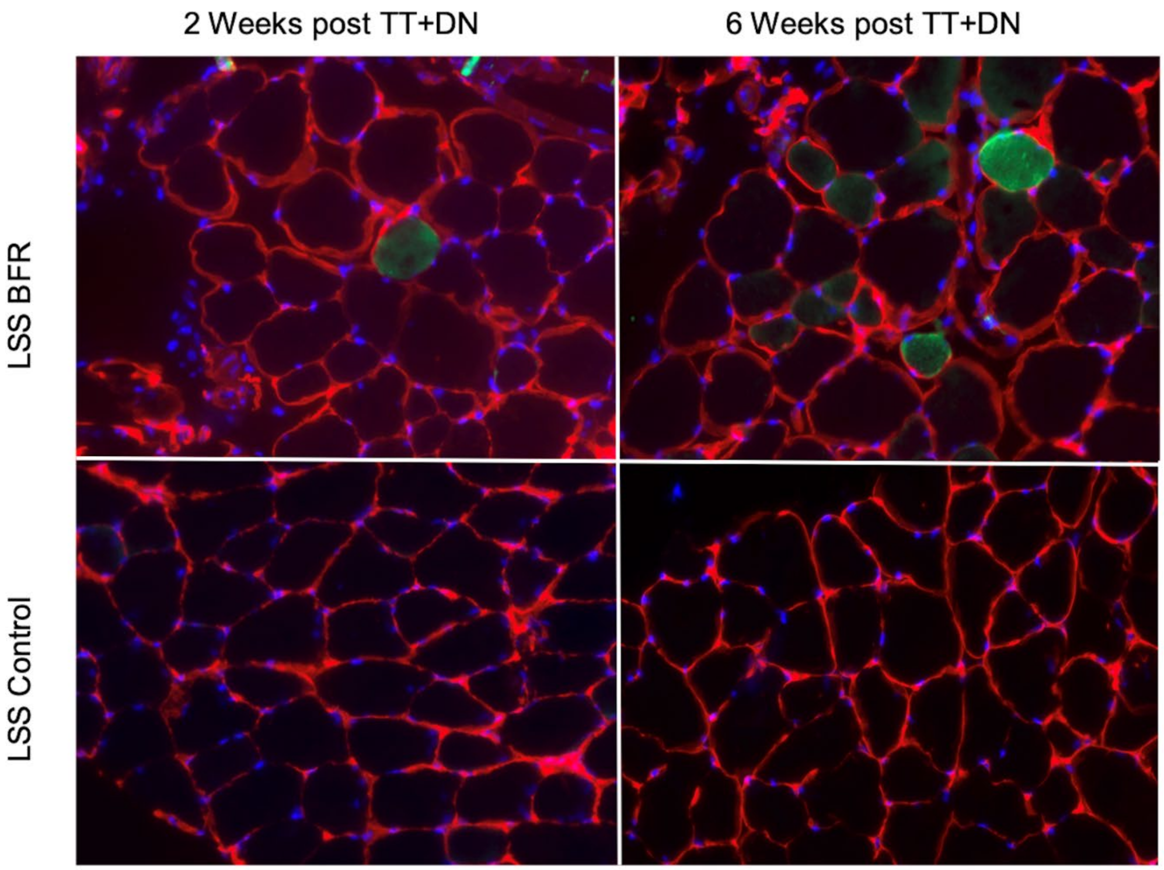

Figure A3.

Representative images of contralateral supraspinatus and infraspinatus muscles from control and BFR-treated mice at 2 and 6 weeks post-TT+DN. MitoTag-GFP$^+$ mitochondria were detected in contralateral muscles of BFR-treated mice, suggesting that BFR may have mild systemic or centrally mediated effects extending beyond the treated limb.
